# Intravenous lidocaine for gut function recovery in colonic surgery: a health economic evaluation of the ALLEGRO randomised clinical trial

**DOI:** 10.1136/bmjopen-2024-088298

**Published:** 2025-02-25

**Authors:** Marek Atter, Andrew Stoddart, Seonaidh Cotton, Thenmalar Vadiveloo, Karen Innes, Angie Balfour, Robert Arnott, Lorna Aucott, Zoe Batham, Irwin Foo, Graeme MacLennan, Susan Nimmo, Doug Speake, John Norrie, Hugh Paterson

**Affiliations:** 1 The University of Edinburgh, Edinburgh, Edinburgh, UK; 2 University of Aberdeen, Aberdeen, UK; 3 Association of Coloproctology of Great Britain and Ireland, London, UK; 4 University of Oxford, Oxford, UK; 5 HSRU, University of Aberdeen, Aberdeen, UK; 6 Western General Hospital, Edinburgh, UK

**Keywords:** Economics, Endoscopy, Clinical Trial, Colorectal surgery

## Abstract

**Objectives:**

To compare costs, health outcomes and cost-effectiveness of using intravenous lidocaine (bolus given at induction of anaesthesia, followed by infusion for 6–12 hours) during colorectal surgery to improve the return of gastrointestinal function.

**Design:**

Within-trial planned analysis of data from a randomised controlled trial using an intention-to-treat approach.

**Setting:**

27 hospitals from across the UK.

**Participants:**

557 patients aged 25–91 having minimally invasive elective colorectal resection.

**Intervention:**

A 1:1 randomisation between intravenous lidocaine and placebo, minimised for age (<50 years, 50–74 years, ≥75 years), gender, and trial centre.

**Primary outcome measures:**

Mean differences between trial arms in 30-day and 90-day quality-adjusted life-years (QALYs) and 30-day total National Health Service costs, as well as the 30-day incremental cost-effectiveness ratio.

**Results:**

Compliance and data quality were high. Intravenous lidocaine is associated with differences of £38 (95% CI: −£463, £589) in total 30-day costs, −0.0005 (95% CI: −0.0027, 0.0015) in 30-day QALYs and −0.0008 (95% CI: −0.0066, 0.0048) in 90-day QALYs. No large, statistically significant or meaningful differences in primary or secondary outcome measures between trial arms were detected, other than for the intervention costs.

**Conclusion:**

Intravenous lidocaine is not found to impact costs or health outcomes for patients undergoing colorectal surgery. In the absence of a clinical effect, disinvestment from perioperative lidocaine could save costs associated with infusion monitoring.

**Trial registration number:**

International Standard Randomised Controlled Trial Number 52352431.

**STRENGTHS AND LIMITATIONS OF THIS STUDY**

- High-quality multicentre data collection, large sample size and low missingness.
- Pragmatic trial design embedded in daily usual practice in sites throughout the UK.
- Parameter uncertainty due to difficulties in sourcing accurate costs of recovery room time.
- Reporting designed for use in future health economic modelling.

## Introduction

 Colorectal surgery is common in UK hospitals. After a segment of the colon is removed and the bowel rejoined, it can take a few days for bowel function (eating and passing flatus and stool) to recover. Almost all patients stay in the hospital until bowel function recovers. In a significant proportion of patients, bowel recovery takes longer than a few days, causing vomiting, abdominal pain and swelling.^1^ These patients are unable to eat until the bowel recovers, requiring supportive treatment (intravenous fluids, etc) and a longer hospital stay. There is no specific treatment to help the bowel recover faster—patients simply have to wait. Modern minimally invasive surgical techniques mean that other aspects of recovery (eg, pain control, resumption of independent mobility) are established within 48 hours. Therefore, interventions able to reduce bowel recovery time have the potential for improved patient comfort, and National Health Service (NHS) cost reduction if earlier discharge can be achieved.

Two small, single-centre randomised controlled trials (RCTs) in elective laparoscopic colonic surgery found that perioperative intravenous lidocaine accelerated bowel function recovery and reduced the length of hospital stay.^2 3^ Lidocaine is cheap, familiar to healthcare workers, widely used and has a well-documented safety profile. Hence, if definitively demonstrated to improve recovery time, it would have a high potential for cost-efficiency.

The ALLEGRO trial tested whether intravenous lidocaine improves recovery of bowel function after minimally invasive elective colorectal resection surgery.^4 5^ The trial found that perioperative administration of 2% intravenous lidocaine infusion did not improve the return of gut function at 72 hours among adults undergoing elective minimally invasive colon resection.^5^

Here, we present the results of the prespecified within-trial economic evaluation for ALLEGRO. The objectives of this analysis were to assess the cost-effectiveness of using intravenous lidocaine, measured in cost per incremental quality-adjusted life-year (QALY), relative to standard care as observed over the 30-day trial period from an NHS and Personal Social Services (PSS) perspective. The data quality also enables us to describe the observed patterns of healthcare utilisation and health utility during the post-surgery recovery.

## Methods

### Trial overview

Full details of the ALLEGRO trial, its procedures (including inclusion/exclusion criteria) and clinical findings can be found in the study protocol and main results paper.^5^ The following summary is included for context only.

ALLEGRO was a multicentre, pragmatic, placebo-controlled, randomised trial. Enrolment occurred from 13 August 2018 to 11 April 2023, with a pause in recruitment from 20 March 2020 through 6 July 2020 due to the SARS-CoV-19 pandemic. The final follow-up was on 10 August 2023. 557 participants from 27 UK hospitals (see online supplementay table 1) undergoing elective colonic resection for colorectal cancer, benign polyps, benign stricture or diverticular disease were randomised on a 1:1 ratio to either^5 6^

- *Intravenous lidocaine:* sterile solution of lidocaine 2% made isotonic with sodium chloride, or
- *Placebo:* 0.9% sterile sodium chloride solution for injection.

An intravenous bolus of 2% lidocaine (or placebo) was administered at induction of anaesthesia over 20 min, followed by intravenous infusion for a minimum of 6 hours up to a maximum of 12 hours. The duration of the infusion was determined preoperatively by the participating units’ normal postoperative availability of continuous cardiac monitoring (mandated during the study as lidocaine toxicity manifests as cardiac arrhythmia). Exact dosing regimens are described in the ALLEGRO trial protocol.^4^

### Economic principles

The methods of calculating costs, health outcomes and cost-effectiveness metrics were outlined in a Health Economic Analysis Plan (HEAP), including preselected price weights, signed off by the lead economist and chief investigator before data lock and unblinding. Our paper follows Consolidated Health Economic Evaluation Reporting Standards guidance for health economic evaluations as summarised in table 1.^7^

**Table 1:** Consolidated Health Economic Evaluation Reporting Standards (2022) checklist

Item	Topic	Page	Section
	*Title*		
1	Title	1	(Title)
	*Abstract*		
2	Abstract	1	Abstract
	Introduction		
3	Background and objectives	2	Introduction
	*Methods*		
4	Health economic analysis plan	3	Trial overview
5	Study population	8	Study population
6	Setting and location	8	Study population
7	Comparators	3	Trial overview
8	Perspective	3	Economic principles
9	Time horizon*	3	Economic principles
10	Discount rate	3	Economic principles
11	Selection of outcomes	5	Estimating outcomes
12	Measurement of outcomes	5	Estimating outcomes
13	Valuation of outcomes	5	Estimating outcomes
14	Measurement and valuation of resources and costs	5	Estimating costs
15	Currency, price date and conversion	5	Estimating costs
16	Rationale and description of model*	12	Discussion
17	Analytics and assumptions	7	Analysis
18	Characterising heterogeneity	7	Analysis
19	Characterising distributional effects	7	Analysis
20	Characterising uncertainty	7	Analysis
21	Approach to engagement with patients and others affected by the study	8	Patient and public engagement
	*Results*		
22	Study parameters	3	Methods
23	Summary of main results	8	Results
24	Effect of uncertainty	11	Cost-effectiveness
25	Effect of engagement with patients and others affected by the study	8	Patient and public engagement
	*Discussion*		
26	Study findings, limitations, generalisability and current knowledge	12	Discussion
	*Other relevant information*		
27	Source of funding	14	Study details
28	Conflicts of interest	14	Study details

* N/A: these items were not required in the analysis (see relevant sections for justification).

To maximise UK policy relevance, the analysis followed National Institute for Health and Care Excellence (NICE) reference specifications.^8^ This included the use of QALYs as the primary health outcome and the adoption of an NHS and PSS perspective with primary cost-effectiveness outcomes presented in cost-utility format in terms of incremental cost-per-QALY.^8^

The time horizon for measuring QALYs and costs and for producing cost-effectiveness results was 30 days. This was the last time point in which both healthcare resource utilisation (HRU) data and patient-reported outcome questionnaires were collected.^4^ An additional 90-day QALY was calculated beyond the 30-day time horizon but was not used in the cost-effectiveness analysis due to the absence of corresponding cost data. As all time horizons were under 1 year, no discounting was necessary for costs or outcomes.

The base year for all analyses was the financial year ending 2022, selected as the latest year for which key price weight sources were available at the time of finalising the HEAP. All analyses were undertaken on an intention-to-treat basis.

### Data collection

Data collection time points were measured in postoperative days (PODs), which included the first 7 days, POD 30 and POD 90 in addition to baseline data (see online supplementay table 2 for a detailed data collection timeline). Primary and secondary care data, along with quality-of-life data, were collected from study questionnaires. Quality-of-life data collection used the validated EQ-5D-5L instrument by EuroQoL, a generic questionnaire comprised of five dimensions: ‘mobility’, ‘self-care’, ‘usual activities’, ‘pain/discomfort’ and ‘anxiety/depression’, each ranked on a Likert scale from 1 (no problems) to 5 (extreme problems).^9^ Other HRU data, consisting of surgery details, adverse events (AEs), complications and length of stay (LOS) were collected from Case Report Forms.

### Estimating outcomes

QALYs were calculated from EQ-5D-5L instrument data.^9^ Data from patient self-reported EQ-5D-5L questionnaires (issued at POD: 1–7, 30 and 90) were converted into health utility scores using a mapping function recommended by NICE. This is a prescored algorithm where each combination of the five domains is allocated a health utility represented by a numerical value, where 0 is equivalent to death and 1 represents full health.^8 10 11^ For each patient, 30- and 90-day QALYs were calculated as a function of health utility scores and PODs using the validated area-under-the-curve formula (using PODs 1, 7, 30 and 90).^12^ Health utility scores from PODs 2–6 were not included in QALY estimates as they were not collected for discharged patients. These were collected opportunistically for context purposes only.

### Estimating costs

Reported healthcare utilisation for each patient was combined with the corresponding price weights (shown in table 2) to estimate cost. Total costs included all costs incurred between the operation and the postoperative questionnaire of day 30.

**Table 2:** Unit costs and price weights

Item	Cost (£)	Source and notes
Surgery costs		
Operation theatre time (1 hour)	1449.34	NHS Scotland annual running costs per theatre (2022/2023) divided by weeks per year and usage time per week (24 hours) (2022/2023), adjusted from £1560.45 to 2022 prices using the ONS health-specific CPI.^14 25^
Recovery room time (1 hour)	69.50	National Cost Collection—Surgical Adult PATIENTS (unspecified specialty, adult critical care, 0 organs supported, service code: CCU02, critical care tab). £1668.02 per bed day (total cost/bed days) divided by 24 to estimate cost per hour.^13^
Ward time (1 hour)	12.21	National Cost Collection—estimated by proxy from an excess bed day (£293 per day/24) for ‘Proximal and Distal Colon Procedures, age≥19’, codes: (FF32A, FF32B, FF32C, FF33A, FF33B), Tabs: ‘APC’ and ‘OPROC’.^13^
Lidocaine (mg)	0.02	BNF.^26^
Intensive care night	2277.65	National Cost Collection—XC05Z: adult critical care, 2 organs supported (choice of supported organs based on clinical advice).^13^
Outpatient attendances		
GP surgery consultations	39.68	PSSRU—GP per patient contact lasting 9.22 min including direct care staff costs with qualification costs. Inflated from £39.23 (2021) to 2022 prices using the ONS health-specific CPI.^14 27^
GP phone consultations	8.77	PSSRU—inflated from £8.67 (2021) to 2022 prices using the ONS health-specific CPI.^14 27^
GP home consultations	117.43	PSSRU—consultation lasting 15 min and 12 min travel time; £4.30 per minute of GP patient contact including direct care staff costs with qualification costs. Inflated from £116.10 (2021) to 2022 prices using the ONS health-specific CPI.^14 27 28^
PN surgery consultations	11.50	Consultation lasting 15.5 min; £44.00 per hour of patient contact including qualification costs. Inflated from £11.37 (2021) to 2022 prices using the ONS health-specific CPI.^14 27 28^
PN phone consultations	7.71	Nurse-led telephone triage based on an average time of 6.56 min. Inflated from £7.62 (2021) to 2022 prices using the ONS health-specific CPI.^14 27^
PN home consultations	20.03	Consultation lasting 15 min and 12 min travel time; £44 per hour of patient contact including qualification costs. Inflated from £19.80 (2021) to 2022 prices using the ONS health-specific CPI.^14 27 28^
DN home consultations	53.74	National Cost Collection—’District Nurse, Adult, Face to face’^13^.
Physiotherapy	100.47	National Cost Collection—’Physiotherapy Service’ (total outpatient attendance).^13^
NHS direct/NHS 24	9.43	PSSRU—inflated from £7.80 (2013) to 2022 prices using the ONS health-specific CPI.^14 27^
Walk-in centre visits	81.93	National Cost Collection—weighted average of HRG codes: VB01Z to VB11Z; service description: ‘NHS Walk in Centres’. Tab: ‘EC’.^13^
Outpatient appointments	165.17	National Cost Collection—’Total Outpatient Attendance’.^13^
Emergency ambulance use	236.81	National Cost Collection—ambulance service (‘Other Currencies’).^13^
ED visits	242.03	National Cost Collection—’Emergency care’.^13^
General surgery	160.62	National Cost Collection—’General Surgery Service’.^13^
Colorectal surgery	130.04	National Cost Collection—’Colorectal Surgery Service’.^13^
GI surgery	169.77	National Cost Collection—’Upper Gastrointestinal Surgery Service’.^13^
Neurology	213.50	National Cost Collection—’Neurology Service’^13^.
Urology	137.74	National Cost Collection—’Urology Service’^13^.
Emergency service	143.74	National Cost Collection—’Emergency Medicine Service’^13^.

BNF, British National Formulary; CPI, Consumer Price Index; DN, district nurse; ED, Emergency Department; GI, gastrointestinal; GP, general practitioner; NHS, National Health Service; ONS, UK Office for National Statistics; PN, practice nurse; PSSRU, Personal Social Services Research Unit.

The hourly cost of operating theatre use published by Public Health Scotland was identified at the review stage after the HEAP was written to replace a previous less robust source.

No specific price weight was located for recovery room time. The cost of the high-dependency unit was used as an approximation following clinical consultation. These are stratified by the number of organs supported (between 0 and 6+) in the National Cost Collection^13^ and 0 organs supported by reasonable approximation. This differs from the cost of intensive care time for which two organs were supported, chosen as a reasonable midway point also guided by clinical advice.

The costs of administering the placebo were excluded as they are not part of the current standard of care.

All unit costs not originally reported in 2022 GBP were converted into the 2022 prices using the Office for National Statistics’ health-specific Consumer Price Index.^14^

### Analysis

Statistical analysis was performed using the R statistical programming language.^15^ For each outcome and cost variable and total costs, the unadjusted arithmetic mean and SD were reported separately for each trial arm, along with the difference in means between arms.

To account for missing data and the non-normal and skewed distribution of estimates, statistical tools from the validated *bootImpute* R programming package were used to estimate cost and QALY 95% CIs for means and differences in means between trial arms.^16^ These combined multiple imputation by chained equations, non-parametric bootstrapping and generalised linear model regression with a gamma distribution and a log link recommended by Manning and Mullahy.^17 18^ For both costs and QALYs, both unadjusted and adjusted results were presented; unadjusted (univariate) results contain the trial arm as the only independent variable, whereas adjusted (multivariate) regression formulas control for age, sex and intravenous lidocaine infusion duration (6 vs 12 hours).

The analysis also included exploratory post hoc observational regression outputs grouping patients by infusion duration (6 vs 12 hours) rather than the trial arm in both adjusted and unadjusted regression formulas.

As per NICE guidelines, the primary cost-effectiveness metric was incremental cost-effectiveness ratios (ICERs) in terms of incremental cost per QALY gained (intervention minus control). These were reported alongside measures of parameter uncertainty in the form of cost-effectiveness plane (CEP) scatterplots and cost-effectiveness acceptability curves (CEACs).^8^ The ICERs and plots were reported separately for unadjusted and adjusted results. The CEPs and CEACs were constructed by calculating incremental costs and QALYs (adjusted and unadjusted) separately for 1000 unpooled simulated data set iterations in the *bootImpute* function, composed of 500 bootstrap samples (*nBoot=500*) each imputed twice (*nImp=2*), as recommended by Hippel and Bartlett.^16^ CEP plots incremental costs against incremental QALYs, whereas the CEAC plots the probability of intravenous lidocaine being cost-effective at various willingness-to-pay (WTP) thresholds by calculating the incremental net monetary benefit for each simulated data set.

### Lidocaine toxicity risk modelling

We initially planned to undertake simplified modelling of longer-term outcomes associated with intravenous lidocaine toxicity (ie, overdose due to incorrect infusion quantity administered by an anaesthetist) such as fatal cardiac arrhythmias. This was not undertaken as the matter was rendered moot by the absence of serious AE (SAE) differences and a clinical benefit to be traded off against.

^5^

## Results

### Study population

Out of 561 participants, 4 participants did not have an operation due to withdrawing before the day of surgery. Only participants who had an operation were included in the health economic analysis. Following the principles of an intention-to-treat analysis, patients who did undergo an operation but did not receive intravenous lidocaine are included in the study. The study population included in the economic evaluation analysis is presented in table 3. Patients were recruited from 27 centres across the UK (see online supplementary table 1).

**Table 3:** Patient population

	Intravenous lidocaine (n=279)n (%)	Placebo (n=278)n (%)
Age (years)		
<50	18 (6.5%)	19 (6.8%)
50–74	198 (71%)	198 (71.2%)
≥75	63 (22.6%)	61 (21.9%)
Range	25–91	33–86
Sex		
Male	157 (56.3%)	151 (54.3%)
Female	122 (43.7%)	127 (45.7%)

### Data quality

The ALLEGRO study is overall characterised by a low degree of missing data (see online supplementary table 3). For health resource use (and subsequently cost categories), the missingness did not exceed 5% of patients, while missingness was below 20% for health utility scores at baseline and PODs 1, 7, 30 and 90. The degree of missingness is higher by design for health utility score data collected between POD 2 and 6 as patients who were discharged were not issued surveys.

12 participants in the intravenous lidocaine arm did not receive lidocaine. This was due to reasons such as investigational medicinal products (IMP) logistics (where the drug was not available to give to the participant) and anaesthetist preference (where the anaesthetist did not want to proceed with the infusion).

**Figure 1:**
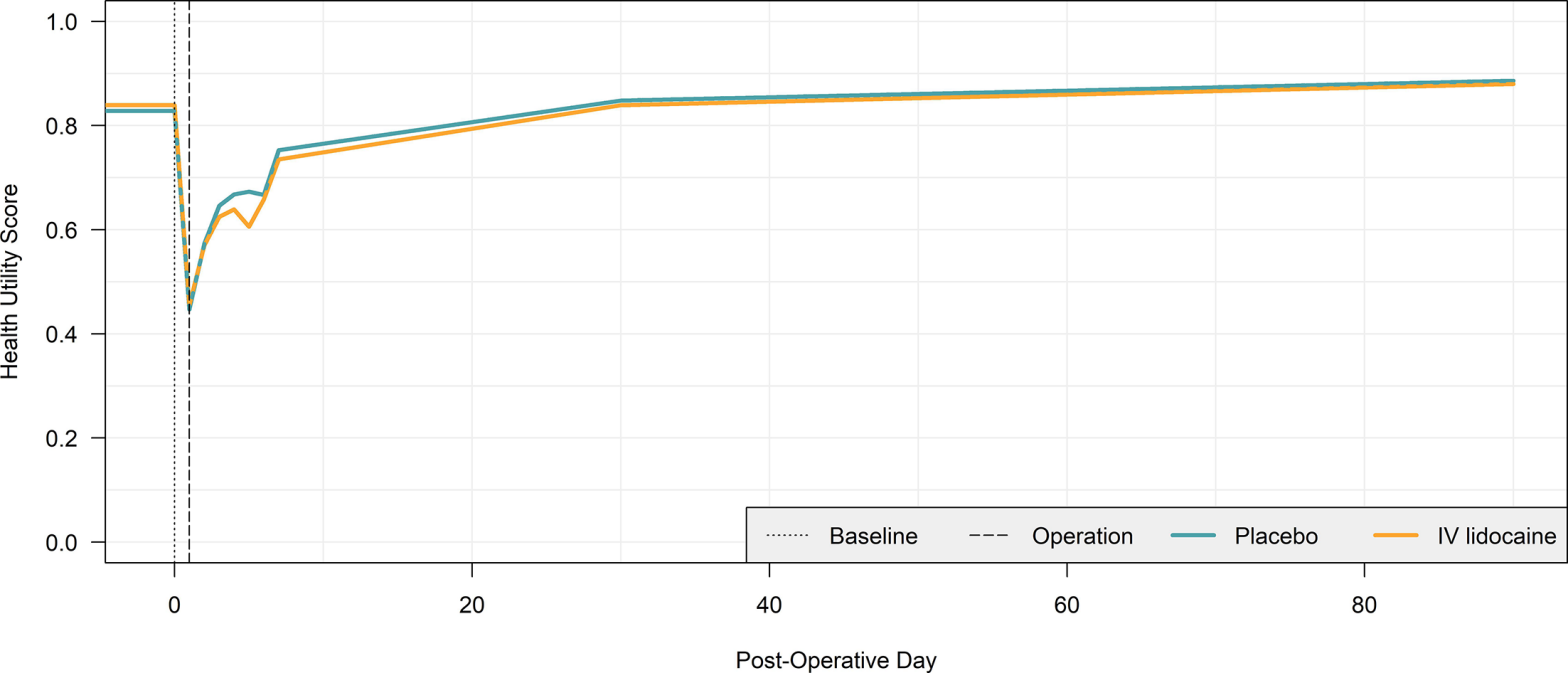
Mean health utility score progression by pos-operative day. IV, intravenous

Table 4 presents the results of the primary health economic outcomes measured in the ALLEGRO trial, including health utilities, QALYs and costs. A breakdown of HRU rates from which costs were calculated is provided in online supplementary table 4. Health utility score progression over time is visualised in figure 1. Table 4 results are univariate, except for the key results of 30-day QALYs and total costs, for which regression-adjusted (multivariate) estimates are provided. All costs in table 4 were measured within a 30-day time horizon.

**Table 4:** Results (means, mean differences, 95% CIs and p values)

Item	Intravenous lidocaine(n=279)	Placebo(n=278)	Difference	95% CI	P value
Health utility					
Health utility (baseline)	0.8348	0.8316	0.0032	(−0.0259, 0.0338)	0.80
Health utility (day 1)	0.4279	0.4746	−0.0467	(−0.0995, 0.0153)	0.15
Health utility (day 2)	0.5636	0.5789	−0.0153	(−0.0707, 0.0246)	0.34
Health utility (day 3)	0.6106	0.6587	−0.0481	(−0.0907, 0.0044)	0.03*
Health utility (day 4)	0.627	0.6806	−0.0536	(−0.0762, 0.0091)	0.12
Health utility (day 5)	0.6241	0.6584	−0.0343	(−0.0610, 0.0395)	0.67
Health utility (day 6)	0.6751	0.6479	0.0272	(−0.0110, 0.0943)	0.12
Health utility (day 7)	0.7305	0.7561	−0.0256	(−0.0561, 0.0049)	0.10
Health utility (day 30)	0.8467	0.8401	0.0066	(−0.0166, 0.0283)	0.61
Health utility (day 90)	0.8926	0.8734	0.0192	(−0.0087, 0.0416)	0.20
QALYs					
QALYs (day 1)	0.0018	0.0018	0	(−0.0001, 0.0001)	0.45
QALYs (day 7)	0.0118	0.0118	0	(−0.0008, 0.0006)	0.84
QALYs (day 30)	0.0604	0.0609	−0.0005	(−0.0027, 0.0015)	0.57
QALYs (day 30, adjusted)	–	–	−0.0007	(−0.0028, 0.0014)	0.51
QALYs (day 90)	0.1993	0.2001	−0.0008	(−0.0066, 0.0048)	0.75
Surgery costs (day 30)					
Operation theatre time	£7424	£7516	−£92	(−£452, £267)	0.58
Recovery room time	£592	£596	−£3	(−£46, £36)	0.76
Ward time	£1620	£1504	£116	(−£99, £346)	0.32
Lidocaine	£18	£0	£18	(£17, £19)	<0.01*
Intensive care night	£287	£246	£41	(−£261, £156 350)	0.70
Total direct surgery cost	£9895	£9857	£38	(−£445, £606)	0.80
Primary care (day 30)					
GP surgery consultations	£8	£14	−£5	(−£9, £0)	0.05
GP phone consultations	£3	£2	£1	(−£0, £1)	0.18
GP home consultations	£2	£3	£0	(−£3, £2)	0.69
PN surgery consultations	£3	£5	−£1	(−£3, £1)	0.30
PN phone consultations	£0	£1	£0	(−£1, £0)	0.22
PN home consultations	£0	£0	£0	(−£1, £0)	0.30
DN home consultations	£26	£26	£0	(−£16, £25)	0.92
Physiotherapy	£1	£0	£1	(−£1, £3)	0.31
NHS direct/NHS 24	£1	£1	£0	(−£0, £0)	0.90
Total primary care cost	£45	£49	−£4	(−£23, £18)	0.55
Secondary care (day 30)					
Walk-in centre visits	£3	£4	−£1	(−£4, £3)	0.51
Outpatient appointments	£77	£72	£5	(−£14, £29)	0.64
Emergency ambulance use	£1	£4	−£4	(−£7, £1)	0.13
ED visits	£13	£15	−£2	(−£10, £10)	0.72
Unplanned admissions	£6	£14	−£8	(−£12, -£1)	0.04*
Total indirect care cost	£100	£106	−£6	(−£33, £21)	0.52
Total costs (day 30)					
Total costs	£10 067	£10 029	£38	(−£463, £589)	0.85
Total costs (adjusted)	–	–	£8	(−£486, £529)	0.95

*Statistically significant at the 95% CI; unadjusted (univariate) results contain trial arm as the only independent variable, whereas adjusted (multivariate) regression formulas control for age, sex and intravenous lidocaine infusion duration (6 vs 12 hours).

day, postoperative day; DN, district nurse; ED, emergency department; GP, general practitioner; NHS, National Health Service; PN, practice nurse; QALY, quality-adjusted life-year.

There was no statistically significant difference in most health utility scores and all cumulative QALYs between trial arms at the 95% confidence level at any time point. The only exception is a small but statistically significant difference of −0.0481 (–0.0907, –0.0044) in the health utility score on POD 3, which had subsided by POD 7. This was not enough to influence the QALY results and may be an artefact of not adjusting for repeated measures.

Differences between trial arms in mean per-patient total costs were not statistically significant at the 95% confidence level. For individual HRU categories, only three items had statistically significant differences:

- *Intravenous lidocaine:* patients in the intravenous lidocaine consumed £18 worth of intravenous lidocaine which was not used in the placebo arm by definition.
- *General practitioner (GP) surgery consultations:* the mean (95% CI) difference in GP surgery consultations is −£5 (−£9, £0) in the intravenous lidocaine arm compared with the placebo arm.
- *Unplanned admissions:* the mean (95% CI) difference in unplanned admission costs is −£8 (−£12, −£1) in the intravenous lidocaine arm compared with the placebo arm.

It should be noted that the detected differences in GP surgery consultation and unplanned admission costs, while technically statistically significant, were negligible. These results should not be subject to overinterpretation as they have not been adjusted for repeated measures and are likely to be artefacts of the heavily skewed nature of cost distributions.

None of the differences in adjusted results presented in table 4 are statistically significant at the 95% confidence level.

### Effect of infusion duration

The post hoc regression analysis shows a statistically significant difference in QALYs at the 95% confidence level, with a decrease in QALYs with shorter infusion times, and a large statistically significant reduction in costs for 6-hour infusion times (see online supplementary table 5). The difference in cost is most likely due to the shorter infusion, by definition, as well as the shorter time in recovery. The QALY difference between 12-hour and 6-hour centres may be confounded by a difference in patient profiles between centres or different perioperative care procedures between centres. These analyses are non-randomised and are intended to be speculative and hypothesis-forming only.

### Cost-effectiveness

The main measure of cost-effectiveness is the ICER. However, in both unadjusted and adjusted results, costs were higher and QALYs lower in the intravenous lidocaine arm; so, the ICERs could not be calculated as intravenous lidocaine is dominated by standard care as a treatment strategy.

**Figure 2:**
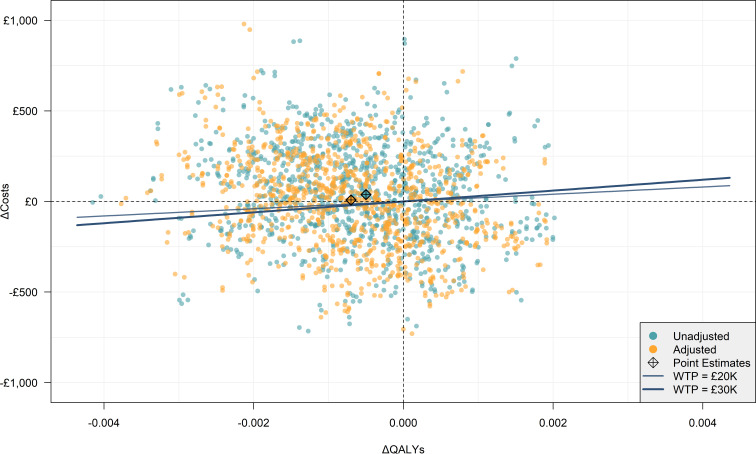
Cost-effectiveness plane. QALY, quality-adjusted life-year; WTP, willingness-to-pay.

Uncertainty around cost-effectiveness estimates was measured using non-parametric bootstrapping and visualised in CEPs in figure 2 for unadjusted and adjusted estimates. Each point on the CEP represents the incremental costs and QALYs (intravenous lidocaine—placebo) of a given simulated bootstrap data set. In both unadjusted and adjusted CEP plots, results span all four quadrants and the base case point is close to the origin. This is expected given the lack of statistically significant differences in mean QALYs and total costs between trial arms.

**Figure 3:**
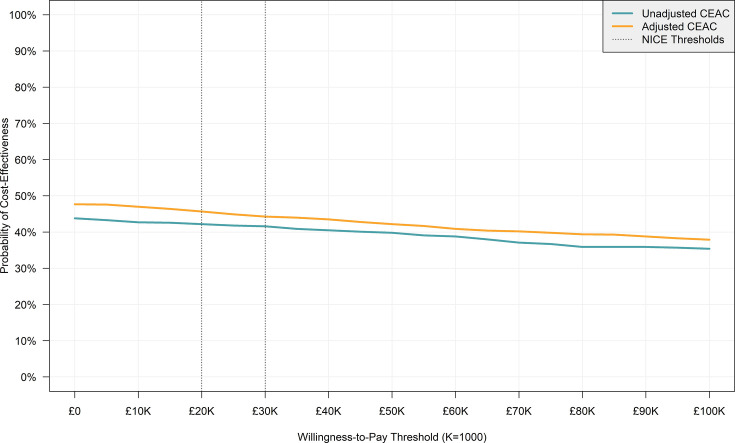
Cost-effectiveness acceptability curve (CEAC). NICE, National Institute for Health and Care Excellence.

The percentage of simulated observations in each CEP quadrant for both adjusted and unadjusted estimates and the percentage of simulated observations under a £20 000 and £30 000 WTP threshold are presented in table 5. Figure 3 presents the CEACs for unadjusted and adjusted results. In both cases, the probability of intravenous lidocaine being cost-effective decreases with the WTP. Nevertheless, the probability is below 50% for both adjusted and unadjusted estimates throughout the WTP, as is expected given the lack of statistically significant differences in mean QALYs and total costs between trial arms.

**Table 5:** Cost-effectiveness results

Adjusted regression	Bootstrapped simulations in each cost-effectiveness plane quadrant (%)				Bootstrapped simulations under WTP threshold (%)	
	**Northwest**	**Northeast**	**Southwest**	**Southeast**	**WTP = £20K**	**WTP = £30K**
No	41.70	14.30	27.50	16.30	42.20	41.60
Yes	41.60	10.70	32.20	15.50	45.70	44.30

WTP, willingness-to-pay.

## Discussion

### Summary

The results of the ALLEGRO health economic analysis are consistent with the clinical results showing no effect of intravenous lidocaine. The differences in total costs and QALYs between placebo and intravenous lidocaine are small and not statistically significant. This was unaffected by a small but statistically significant difference in the health utility score on POD 3 (see table 4), which itself is likely an artefact of not adjusting for repeated measures. Visualisation of uncertainty of the CEP plots shows a spread of simulated cost-effectiveness results across the CEP quadrants, confirming that there is likely no difference between trial arms other than random chance.

Interpretation of the results should account for the relative importance of key price weights in determining total costs and cost differences. The low cost of the IMP (~£18 per patient) rendered its contribution to total costs overwhelmed by the noise of a wide range of non-significant but higher-cost HRU factors (eg, unscheduled assessments).

Our post hoc analysis did, however, find a statistically significant improvement in both costs and QALYs of centres with a 6-hour infusion duration (of either IMP or placebo) over those of 12 hours, irrespective of the trial arm. However, since the results of this study point to lidocaine having no benefit over standard care, the duration of the infusion is therefore rendered irrelevant. Furthermore, we advise caution in the interpretation of these results as they were not randomised and do not account for differences in patient populations or surgeon experience between sites. We include these as potentially hypothesis-forming outputs for future research considering other infusions only.

The decision not to proceed with building the model was based on the following two considerations: (1) simulating the effects of low-frequency and high-impact events was deemed unnecessary given a lack of difference in SAE rates between trial arms, and (2) simulating long-term costs and outcomes of using intravenous lidocaine following colorectal surgery was deemed unnecessary given no statistically significant difference in costs and outcomes between arms within the trial time horizon.

### Strengths and limitations

The strengths of the ALLEGRO economic evaluation lie in the detailed data collection, both for quality of life and cost outcomes, with relatively low missingness (see online supplementary table 3). HRU variables (and thus costs) are characterised by low missingness (<10%) for both arms. The EQ-5D-5L-derived health utility score is a composite measure that depends on multiple elements of the patient questionnaire and thus has higher missingness. Health utility score missingness is higher for PODs 2–6 (as they were only completed in-hospital so people who had been discharged did not have the opportunity to complete them), which are excluded from QALY calculations, and lower (<20%) for PODs 1, 7, 30 and 90. Data quality further benefits from the size of the trial, the broad range of hospital contexts included and high compliance.^5^

A further advantage of ALLEGRO is that it is a relatively unique study as there is limited health economic literature related to lidocaine for colorectal surgery recovery. A rapid (non-systematic) PubMed literature search was conducted to search relevant literature on the use of lidocaine or alvimopan (a drug found in RCTs to improve bowel recovery) in colorectal surgery, which identified two protocols for lidocaine RCTs (one of which is ALLEGRO) but no complete RCTs, and four observational studies related to alvimopan.^419^,^24^ In contrast to the results of lidocaine’s effect in this study, alvimopan is reported to reduce LOS and costs following colorectal surgery.^22^

The health economic analysis of ALLEGRO shares the limitations of its main clinical analysis, which include a lack of information about participant race, ethnicity or socio-economic status, and the exclusion of more complex colorectal operations (eg, low rectal cancer).^5^

The cost results are further subject to a degree of parameter uncertainty (see table 2). For example, the hourly cost of the recovery room was estimated by proxy, but the difference in costs was small and statistically insignificant. In this case, more accurate price weights are unlikely to meaningfully change the results of our analysis. While we acknowledge this limitation of the study, future research in this area should consider micro-costing the recovery room time and procedure to produce more accurate cost parameters and results.

## Conclusion

While both the clinical trial and the health economic analysis show no effect of intravenous lidocaine, this article is an important contribution to the literature due to the robustness of the trial warranting more definitive statements that lidocaine does not affect gut recovery in this patient population. We hope our results will counteract publication bias that may help guide future research funding. Similarly, with low missingness, the publication of HRU results and reported health utility scores in this article can be a potentially very useful source of parameter estimates to aid future modelling of postoperative colorectal surgery, and we have included details within our results tables to aid such reuse.

We present robust data strongly indicating intravenous lidocaine is not found to impact costs or health outcomes for patients undergoing colorectal surgery, other than the lidocaine infusion itself. In the absence of clinical effects, disinvestment from perioperative lidocaine could save costs associated with infusion monitoring. Future research may wish to focus on alternative strategies for the return of gut function and duration of recovery time.

## Supplementary material

10.1136/bmjopen-2024-088298 online supplemental file 1

## Data Availability

Patient data have been managed to safeguard the confidentiality of patients, consistent with the terms of consent signed by patients. For data requests pertinent to the main clinical analysis, see online supplement 4 of the ALLEGRO clinical paper.^5^ All data requests specific to the health economic analysis should be submitted by the Edinburgh Clinical Trials Unit (ECTU, email: ECTUdatashare@ed.ac.uk) for consideration. Access to anonymised data may be granted following review. Any data sharing approved would have to ensure patient confidentiality.
