# Supplementary material for: Intravenous lidocaine for gut function recovery in colonic surgery: a health economic evaluation of the ALLEGRO randomised clinical trial
Source: BMJ Open. 2025 Feb 25;15(2):e088298. doi: 10.1136/bmjopen-2024-088298 (PMC12083329; doi:10.1136/bmjopen-2024-088298)
Supplement: online supplemental file 1 [file bmjopen-15-2-s001.pdf]

Intravenous Lidocaine for Gut Function Recovery in Colonic Surgery: A Health Economic Evaluation of the ALLEGRO Randomised Clinical Trial

SUPPLEMENTARY MATERIAL

Supplementary Table 1: Patient Recruitment by Centre

**Centre**	**IV lidocaine (N = 279)** n (%)	**Placebo (N = 278)** n (%)
Edinburgh	55 (19.7%)	57 (20.5%)
Royal Blackburn Hospital	4 (1.4%)	5 (1.8%)
Rotherham Hospital	5 (1.8%)	2 (0.7%)
Royal Berkshire Hospital	24 (8.6%)	23 (8.3%)
Norfolk and Norwich	4 (1.4%)	8 (2.9%)
Medway Maritime Hospital	28 (10%)	27 (9.7%)
Belfast City Hospital	6 (2.2%)	6 (2.2%)
Newcastle Royal Infirmary	7 (2.5%)	7 (2.5%)
James Cook	3 (1.1%)	4 (1.4%)
University Hospital of North Tees	2 (0.7%)	3 (1.1%)
Yeovil	4 (1.4%)	5 (1.8%)
Wales	17 (6.1%)	17 (6.1%)
Broomfield Hospital	13 (4.7%)	12 (4.3%)
Queen Elizabeth University Hospital	12 (4.3%)	9 (3.2%)
Arrowe Park Hospital	8 (2.9%)	8 (2.9%)
Addenbrooke's Hospital	17 (6.1%)	15 (5.4%)
St Georges Hospital	16 (5.7%)	17 (6.1%)
Peterborough	1 (0.4%)	2 (0.7%)
Birmingham - QEH	2 (0.7%)	1 (0.4%)
Charing Cross	2 (0.7%)	2 (0.7%)
Worcestershire Royal Hospital	5 (1.8%)	6 (2.2%)
Leeds	24 (8.6%)	22 (7.9%)
RAH - Paisley	6 (2.2%)	5 (1.8%)
Dorset	1 (0.4%)	2 (0.7%)
Nth Bristol	3 (1.1%)	4 (1.4%)
Torbay	7 (2.5%)	6 (2.2%)
Royal Devon and Exeter	3 (1.1%)	3 (1.1%)

Supplementary Table 2: Data Collection Timepoints

POD*	EQ-5D-5L	HRU*
≤0 (Baseline)	✓	x
1	✓	✓ (surgery details & AEs*)
2-7	✓	✓ (AEs)
Discharge	x	✓ (complications & LOS*)
30	✓	✓ (primary & secondary care)
90	✓	x

**AEs – Adverse Events, HRU – Healthcare Resource Use, LOS – Length of Stay, POD – Post-Operative Day*

Supplementary Table 3: Patients with Missing Data

**Variable**	**IV Lidocaine (N=279)** n (%)	**Placebo (N=278)** n (%)
**Health Utility**		
Health Utility (Baseline)	7 (2.5%)	14 (5%)
Health Utility (POD* 1)	39 (14%)	44 (15.8%)
Health Utility (POD 2)	53 (19%)	51 (18.3%)
Health Utility (POD 3)	81 (29%)	75 (27%)
Health Utility (POD 4)	133 (47.7%)	138 (49.6%)
Health Utility (POD 5)	184 (65.9%)	188 (67.6%)
Health Utility (POD 6)	209 (74.9%)	219 (78.8%)
Health Utility (POD 7)	39 (14%)	32 (11.5%)
Health Utility (POD 30)	23 (8.2%)	18 (6.5%)
Health Utility (POD 90)	31 (11.1%)	31 (11.2%)
**HRU: Operation**		
Operation Theatre Time	0 (0%)	2 (0.7%)
Recovery Room Time	8 (2.9%)	7 (2.5%)
Ward Time	9 (3.2%)	8 (2.9%)
Lidocaine	13 (4.7%)	0 (0%)
Intensive Care Night	1 (0.4%)	0 (0%)
**HRU: Primary Care**		
GP* Surgery Consultations	6 (2.2%)	10 (3.6%)
GP Phone Consultations	6 (2.2%)	11 (4%)
GP Home Consultations	6 (2.2%)	11 (4%)
PN* Surgery Consultations	6 (2.2%)	10 (3.6%)
PN Phone Consultations	6 (2.2%)	11 (4%)
PN Home Consultations	6 (2.2%)	11 (4%)
DN* Home Consultations	7 (2.5%)	10 (3.6%)

Variable	IV Lidocaine (N=279)<br>n (%)	Placebo (N=278)<br>n (%)
Physiotherapy	6 (2.2%)	11 (4%)
NHS* Direct/NHS 24	6 (2.2%)	11 (4%)
**HRU: Secondary Care**		
Walk-in Centre Visits	6 (2.2%)	10 (3.6%)
Outpatient Appointments	5 (1.8%)	8 (2.9%)
Emergency Ambulance Use	6 (2.2%)	10 (3.6%)
ED* Visits	3 (1.1%)	7 (2.5%)
Unplanned Admissions	1 (0.4%)	1 (0.4%)
**Cost Totals**		
Total Direct Surgery Cost	14 (5%)	10 (3.6%)
Total Primary Care Cost	7 (2.5%)	11 (4%)
Total Indirect Secondary Care Cost	6 (2.2%)	10 (3.6%)
Total Cost	19 (6.8%)	16 (5.8%)

**DN – District Nurse, ED – Emergency Department, GP – General Practitioner, NHS – National Health Service, PN – Practice Nurse, POD – post-operative day*

Supplementary Table 4: Summary of Observed HRU

Resource	**IV lidocaine** (N = 279)<br>Mean ± SD	**Placebo** (N = 278)<br>Mean ± SD
**Surgery**		
Operation Theatre Time (hours)	5.1 ± 1.4	5.2 ± 1.4
Recovery Room Time (hours)	8.5 ± 3.6	8.6 ± 3.6
Ward Time (hours)	132.7 ± 125.4	123.1 ± 91
Lidocaine (mg)	912.3 ± 383.9	0 ± 0
Intensive Care Night	0.1 ± 0.8	0.1 ± 0.6
**Primary Care**		
GP* Surgery Consultations	1.8 ± 0.4	1.8 ± 0.4
GP Phone Consultations	1.8 ± 0.4	1.8 ± 0.4
GP Home Consultations	2 ± 0.1	2 ± 0.1
PN* Surgery Consultations	1.8 ± 0.4	1.8 ± 0.4
PN Phone Consultations	2 ± 0.2	1.9 ± 0.2
PN Home Consultations	2 ± 0.1	2 ± 0.1
DN* Home Consultations	1.9 ± 0.3	1.9 ± 0.3
Physiotherapy (sessions)	2 ± 0.1	2 ± 0.1
NHS* Direct/NHS 24 (calls)	1.9 ± 0.2	1.9 ± 0.2
**Secondary Care**		

Resource	**IV lidocaine** (N = 279)<br>Mean ± SD	**Placebo** (N = 278)<br>Mean ± SD
Walk-in Centre Visits	2 ± 0.2	2 ± 0.2
Outpatient Appointments	1.6 ± 0.5	1.7 ± 0.5
Emergency Ambulance Use	2 ± 0.1	2 ± 0.1
ED* Visits	1.9 ± 0.2	1.9 ± 0.2
Unplanned Admissions	0 ± 0.3	0.1 ± 0.3

**DN – District Nurse, ED – Emergency Department, GP – General Practitioner, NHS – National Health Service, PN – Practice Nurse*

Supplementary Table 5: Effect of Shorter Infusion Duration on Outcomes (6 hours – 12 hours)

Variable	**Unadjusted**<br>Mean (95% CI)	**P**	**Adjusted**<br>Mean (95% CI)	**P**
QALYs†	-0.0037 (-0.0059, -0.0014)	<0.01*	-0.0035 (-0.0057, -0.0013)	<0.01*
Total Costs	-£824 (-£1,296, -£328)	<0.01*	-£732 (-£1,164, -£279)	<0.01*

†QALY – Quality-Adjusted Life Year; [*] – statistically significant at the 95% confidence level
