# Supplementary material for: Intravenous lidocaine for gut function recovery in colonic surgery: a health economic evaluation of the ALLEGRO randomised clinical trial
Source: BMJ Open. 2025 Feb 25;15(2):e088298. doi: 10.1136/bmjopen-2024-088298 (PMC12083329; doi:10.1136/bmjopen-2024-088298)
Supplement: Supplementary file 2 [file bmjopen-2024-088298.reviewer_comments.pdf]

PEER REVIEW HISTORY

BMJ Open publishes all reviews undertaken for accepted manuscripts. Reviewers are asked to complete a checklist review form and are provided with free text boxes to elaborate on their assessment. These free text comments are reproduced below.

ARTICLE DETAILS

Title (Provisional)

Intravenous Lidocaine for Gut Function Recovery in Colonic Surgery: A Health Economic Evaluation of the ALLEGRO Randomised Clinical Trial

Authors

Atter, Marek; Stoddart, Andrew; Cotton, Seonaidh; Vadiveloo, Thenmalar; Innes, Karen; Balfour, Angie; Arnott, Robert; Aucott, Lorna; Batham, Zoe; Foo, Irwin; MacLennan, Graeme; Nimmo, Susan; Speake, Doug; Norrie, John; Paterson, Hugh

---

VERSION 1 - REVIEW

**Reviewer**	**1**
**Name**	**Dale, Megan**
**Affiliation**	**Cardiff and Vale University Health Board**
**Date**	**11-Sep-2024**
**COI**	**None**

I enjoyed reading this interesting and clearly presented manuscript. I have only a couple of points of clarification, or queries.

Firstly, have you considered using the NHS Scotland theatre costs published by Public Health Scotland at https://publichealthscotland.scot/publications/scottish-health-service-costs/scottish-health-service-costs-summary-for-financial-year-2022-to-2023/files-listing-2022-to-2023/ It is however unlikely to make a meaningful difference to the conclusions.

I noted that the mapping to EQ-5D-3L value set was referenced to Hernández Alava et al. 2023. The NICE process and methods [PMG36] manual currently states "The mapping function developed by the Decision Support Unit (Hernández Alava et al. 2017), using the 'EEPRU dataset' (Hernández Alava et al. 2020), should be used for reference-case analyses." These are all related methods and publications, however some clarification would be helpful.

Supplementary table 4 - there are some costs that may not be correctly referenced / described:

Physiotherapy is listed as £118.88 from PSSRU 2021, however it is not present in that year, there are costs for Physiotherapy in community services of £67 per group session? Also available for 2022 as £92 per session.

Also, could you state in the table that the general surgery through to emergency medicine items are for outpatient attendances - just for clarity.

**Reviewer**	**2**
**Name**	**Narayan, Raja**
**Affiliation**	**Dana-Farber Cancer Institute**
**Date**	**16-Sep-2024**
**COI**	**None**

The authors present the health quality and economic results for a recent, robust, randomized placebo-controlled, double-blinded trial investigating return of bowel function related to the use of 6 and 12hr perioperative IV lidocaine for minimally invasive colorectal surgery. The authors should be congratulated for accomplishing this trial among 27 centers through the coronavirus pandemic. The negative results of this trial are further supported by this health quality and cost analysis essentially showing no difference between the arms. A few minor questions remain regarding these findings:

- Although the authors note that the estimated cost for operating theatre use was approximated in the Methods, this should really be included in a Limitations section of the Discussion for completeness.

- The Limitation section is missing and should be added as it is a component of the CHEERS checklist.

- From review of the available data in the clinical trial, it is surprising that the length of stay was nearly a week in both arms given that half the patients underwent minimally invasive right colectomy. There is no specific detail provided on parameters for discharge (e.g. 24 hours on oral analgesics, need for bowel movement vs passage of flatus alone, etc). This should be provided in the clinical trial at a minimum with reference to this in the article in question for this review.

- There is no reference to whether opiate differed between patients in either arm and whether persistent opiate or other analgesic use may be a factor in health quality at POD30 or 90. This may shed additional light on the utility or futility of incorporating perioperative IV lidocaine in this context.

**Reviewer**	**3**

Name Dack, Kyle

Affiliation University of Bristol, MRC Integrative Epidemiology Unit

Date 15-Oct-2024

COI None

This manuscript reports the absence of an association between IV lidocaine during colorectal surgery and health outcomes/costs, a finding is of high clinical value and scientific novelty.

The statistical methodology is sound, and results are reported accurately. I have a few minor suggestions to add more information:

Introduction

1. P4 lines 6-21. I suggest breaking this into two paragraphs, one to introduce the significance of colorectal surgery recovery times, and the second to summarise the evidence. It would also be helpful to readers if the results from two previous ALLEGRO papers were summarised in this second paragraph.

Methods

2. Please add the dates of the trial to the study overview or data collection section.

3. P4 line 39. The full study procedures are reported to be available in reference 5, but this is currently unpublished. If this reference is not likely to be available by the time the present paper is published, the authors could consider referencing the protocol instead, uploading reference 5 as a preprint, or including more details in the present paper.

4. Supplementary table 1. As a suggestion only, the authors may wish to double-check that this table conforms to any relevant data policies from the participating trial centres. I am aware that for many cohorts, small numbers (e.g. n<5) are required to be censored.

5. P5 line 37. Please include an explanation of what is EQ-5D-5L data and how it links to the outcomes of interest.

6. P6 lines 13-14. Given that it is discussed quite extensively, I suggest adding the reference for where the hourly cost of operating theatre use was identified.

Results

7. It would be helpful to include one or two lines summarising the data quality as shown in supplementary table 5, e.g. excluding health utility surveys, all measures were missing in less than 5% of cases.

8. Table 2. This appears to be the first mention of Health Utility as a variable in the main paper. In the methods it is referred to as health state utility and utility scores. I suggest clarifying this in the methods and explaining in more detail what the score is designed to

represent. Additionally, are the number of decimal places meaningful or could they be reduced?

9. P10 line 10. This line refers to table 2, but this does not seem relevant – table 2 reported univariate results. Please add adjusted regression results in the main or supplementary materials.

VERSION 1 - AUTHOR RESPONSE

Dear Dr Megan Dale,

Thank you for reviewing our manuscript and for your points of clarification. Your suggestions have been immensely helpful, so please find the following points addressed:

1. The NHS Scotland theatre costs are a much better source than Fletcher (2017). I re-ran the analysis with the new price weight. While the NHS Scotland cost was similar to Fletcher when adjusted to a common base year (£1,449 vs £1,356) and had little impact on results, the change has made our analysis much more robust. Please see the updated unit cost table (now Table 1).
2. I have amended the EQ-5D-3L mapping reference to NICE [PMG36] and Hernández Alava et al. (2020) instead of 2024 and corrected the "estimating outcomes" section.
3. You correctly pointed out a mistake in referencing physiotherapy. For consistency, this has been updated with the National Cost Collection 2021/22 reference cost of £100 for "physiotherapy service" – please see the updated unit cost table (now Table 1) and results.
4. Following your suggestion, I have updated the unit cost table with headings, split into "Surgery Costs" and "Outpatient Attendances" for clarity.

Dear Dr Raja Narayan,

Thank you for reviewing our paper. I am very grateful for your positive feedback and comments. Please find the response to the points you raised:

1. The operating theatre cost has been updated, following Dr Dale's suggestion. This thankfully renders the need for it as a limitation moot, though you were correct it would have warranted mention.
2. Following your suggestion, please see a new "strengths and limitations" section included in the Discussion.
3. More information on length of stay and perioperative care practices is now available in the published main clinical paper (doi:10.1001/jama.2024.23898) – please see the updated reference. We have chosen not to detail clinical factors like this explicitly in the economic paper, as we feel that is outside of its scope.
4. The main paper contains data on early opiate analgesia consumption (within 24 hours of surgery). We didn't collect data on opioid consumption at the later time points as we were looking for an early signal. In UK practice it would be extremely unusual to continue opioid analgesia after minimally invasive colectomy beyond 7-10 days.

Dear Dr Kyle Dack,

Thank you for your review of our paper and your comments on its clinical value and novelty. Please find our response to your comments and feedback:

1. Following your suggestions, we have updated the Introduction section.
2. Please see the updated “trial overview” section which includes the dates of the trial from enrolment to follow-up.
3. We have now updated the reference with the published clinical paper for ALLEGRO (doi:10.1001/jama.2024.23898)
4. We have referred your query on supplementary table 1 to the ALLEGRO chief investigator who confirmed that the table does not need to be censored, so we decided not to edit it.
5. We have added a description of the EQ-5D-5L instrument in the “estimating outcomes” section.
6. The operating theatre cost has been updated, following Dr Dale’s suggestion.
7. Following your suggestion, we have added a few lines of summary in the “data quality” section.
8. Following your suggestion, we have standardised references to health utility scores. We also agree that having 4 decimal places is mostly overkill, but since QALY differences are our most important outcome and are measured in increments of 0.0001s, we chose to have 4 decimal places for health utility and QALYs for consistency and comparison.
9. Adjusted regression results are present in the main results table, but only for key results (day 30 QALYs and total costs). This is now clarified in the text and table footnote.

VERSION 2 - REVIEW

**Reviewer**	**1**
**Name**	**Dale, Megan**
**Affiliation**	**Cardiff and Vale University Health Board**
**Date**	**21-Dec-2024**
**COI**	

Thank you for your changes, and comments.
